# Preventing Life-Threatening Hyperglycemia in Immune Checkpoint Inhibitor-Induced Type 1 Diabetes: Insights From Two Cases and Literature Review

**DOI:** 10.7759/cureus.91593

**Published:** 2025-09-04

**Authors:** Nako Matsumoto, Hitoshi Iwasaki, Yuhei Sasai, Nao Aono-Soma, Motohiro Sekiya

**Affiliations:** 1 Department of Endocrinology and Metabolism, University of Tsukuba, Tsukuba, JPN

**Keywords:** fulminant type 1 diabetes, immune checkpoint inhibitors, immune checkpoint inhibitors-induced type 1 diabetes, immune-related adverse events, red cell distribution width

## Abstract

In recent years, the use of immune checkpoint inhibitors (ICIs) has expanded rapidly, accompanied by a marked increase in associated immune-related adverse events (irAEs). Among these, ICI-related type 1 diabetes (ICI-T1D) is frequently recognized only after the onset of life-threatening hyperglycemia, in part due to its rare prevalence and latent nature of disease progression. Herein we report two cases of ICI-T1D successfully treated before the development of marked hyperglycemia. In both cases, despite the complete depletion of insulin secretion, blood glucose control was achieved prior to the onset of diabetic ketoacidosis, and the clinical course could be monitored in detail, making them valuable cases for documentation. Taken together with the literature, these cases suggest that, compared with classic fulminant type 1 diabetes (FT1D), ICI-T1D tends to progress more gradually. Even in the presence of only mild hyperglycemia and partially preserved insulin secretion, its onset should be suspected and the clinical course monitored carefully. Since most patients undergo regular blood testing, careful monitoring may allow for the timely detection and optimal management of ICI-T1D. In addition, we found that not only glucose metrics but also transient increases in red cell distribution width (RDW) levels may be a new predictive marker for irAEs.

## Introduction

In recent years, immune checkpoint inhibitors (ICIs) have become increasingly valuable in the treatment of malignant tumors [1,2], and their use is steadily expanding. However, this has been accompanied by a rise in immune-related adverse events (irAEs), an unavoidable consequence of ICI therapy, with various endocrine cells being among the most common targets [3]. Among these, ICI-related type 1 diabetes (ICI-T1D) is characterized by the complete loss of insulin secretion, and delayed diagnosis can result in life-threatening conditions, such as diabetic ketoacidosis, underscoring the need for vigilance [4,5]. Nevertheless, there is little consensus on optimal patient monitoring, and there is an urgent need for reliable biomarkers or novel indicators to facilitate timely detection, considering the latent nature of progression and rare incidence.

A similarly acute-onset condition characterized by the rapid depletion of insulin secretion is fulminant type 1 diabetes (FT1D) [6]. The diagnostic criteria, as proposed by the Japan Diabetes Society, include (1) the abrupt onset of hyperglycemic symptoms with markedly elevated plasma glucose levels and near-normal glycated hemoglobin (HbA1c), (2) evidence of ketoacidosis at first presentation, and (3) almost complete loss of endogenous insulin secretion, as indicated by low or undetectable serum C-peptide levels [7]. FT1D is often triggered by antecedent infections; however, the precise etiology remains largely unknown. In general, autoantibodies, such as anti-glutamic acid decarboxylase (GAD) antibody, are negative in FT1D, whereas ICI-T1D can present with either positive or negative autoantibody status, representing a point of distinction between the two entities [8]. Certain human leukocyte antigen (HLA) types have been reported to confer increased susceptibility to FT1D, some of which also overlap with those associated with ICI-T1D [9]. This suggests that the two diseases may share some underlying molecular pathways while possessing distinct mechanisms.

In addition, the programmed cell death protein-1 (PD-1)/programmed death ligand-1 (PD-L1) axis has been recognized as being closely linked to aging. Aging is associated with increased PD-L1 expression in various cell types [10], and ICI therapy has been reported to exert anti-aging effects through the elimination of PD-L1-expressing senescent cells (senolysis) [11]. Furthermore, a study elucidating the detailed molecular basis of caloric restriction in rodents identified red cell distribution width (RDW) as a potential biomarker of aging [12], which was supported by recent clinical studies [13]. Since RDW is a hematological parameter reflecting the variations of red blood cell volume, it is influenced by factors affecting erythropoiesis, such as inflammation, oxidative stress, nutritional conditions, and so on. Perhaps due to this broad-range sensitivity, RDW emerges as a biomarker for multiple diseases [14,15].

Here, we show two cases of ICI-T1D successfully treated without developing critical hyperglycemia. These cases provide valuable clues to optimize our patient monitoring strategies.

## Case presentation

Case 1

A 75-year-old Japanese woman was diagnosed with stage IVB lung large cell neuroendocrine carcinoma, and treatment with atezolizumab (anti-PD-L1 antibody) was initiated the following month. Two months later, she was referred to our department since her routine blood tests revealed hypothyroidism. She was diagnosed with ICI-related hypothyroidism. The response to atezolizumab was assessed as a partial response (PR), and atezolizumab was continued with levothyroxine supplementation.

**Figure 1:**
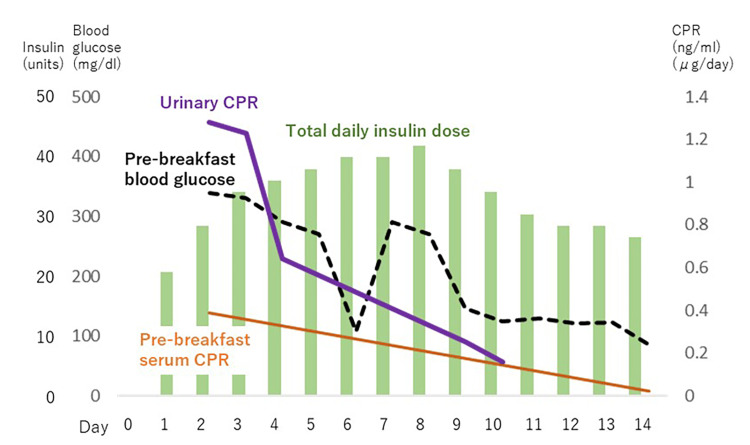
Time course of blood glucose levels and endogenous insulin secretion (Case 1) Day 1 is the day of admission. Blood glucose levels were markedly elevated, but improved rapidly following the initiation of insulin therapy. Serum C-peptide (CPR) and urinary C-peptide were already depleted by day two, and both continued to decline over time, eventually reaching complete depletion.

She had no history of diabetes mellitus, with her HbA1c ranging from 5%, and random blood glucose levels were typically between 80 and 100 mg/dL. However, five months after initiation of atezolizumab, her random blood glucose was elevated to 273 mg/dL with HbA1c of 5.2%. Although neither ketonuria nor symptoms of hyperglycemia were present, her immunoreactive insulin (IRI) level was reduced to 3.6 μU/mL. Since the possibility of fulminant type 1 diabetes associated with atezolizumab could not be ruled out, she started to monitor her blood glucose levels with a personal device. The following morning, her blood glucose was further increased to 491 mg/dL. She was promptly admitted for further evaluation. Her laboratory findings before admission are summarized in Table 1, and her inpatient clinical course is illustrated in Figure 1.

**Table 1:** Laboratory data (Case 1) AST: Aspartate Aminotransferase, ALT: Alanine Aminotransferase, LDH: Lactate Dehydrogenase, BUN: Blood Urea Nitrogen, FT3: Free Triiodothyronine, FT4: Free Thyroxine, TSH: Thyroid-Stimulating Hormone, IRI: Immunoreactive Insulin, GAD Antibody: Anti-Glutamic Acid Decarboxylase Antibody, IA-2 Antibody: Anti-Insulinoma-Associated Antigen-2 Antibody, ZnT8 Antibody: Anti-Zinc Transporter 8 Antibody, HLA: Human Leukocyte Antigen

Test	Result	Reference range
White blood cells (/μL)	4000	4000-9000
Hemoglobin (g/dL)	9.7	12-16
Platelet (10^4^/μL)	20.6	15-35
AST (U/L)	13	8-38
ALT (U/L)	7	4-44
LDH (U/L)	164	124-222
Amylase (U/L)	67	40-126
Lipase (mg/dL)	18	13-42
BUN (mg/dL)	14	8-20
Creatinine (mg/dL)	0.71	0.47-0.79
Sodium (mEq/L)	143	135-147
Chloride (mEq/L)	106	98-108
Potassium (mEq/L)	5.2	3.6-5.0
FT3 (pg/mL)	1.8	2.3-4.0
FT4 (ng/dL)	0.7	0.9-1.7
TSH (μIU/mL)	59.6	0.5-5.0
HbA1c (%)	5.2	4.6-6.2
Glycoalbumin (%)	17.6	11-16
Plasma glucose (mg/dL)	273	70-199
IRI (μU/mL)	3.6	1.1-17
GAD antibody (U/mL)	<5.0	<5.0
IA-2 antibody (U/mL)	<0.6	<0.6
ZnT8 antibody (U/mL)	<10.0	<10.0
Pre-breakfast plasma glucose (mg/dL)	339	
Post-breakfast plasma glucose (mg/dL)	345	
Pre-breakfast serum C-peptide (ng/mL)	0.39	
Post-breakfast serum C-peptide (ng/mL)	0.42	
Urine protein	1+	
Urine glucose	2+	
Urine ketone bodies	Negative	
Urinary C-peptide (μg/day)	1.28	
HLA	DRB1 09:01:02/13:02:01 DQB1 06:04:01/03:03:02 DPB1 04:01:01/05:01:01	

**Figure 2:**
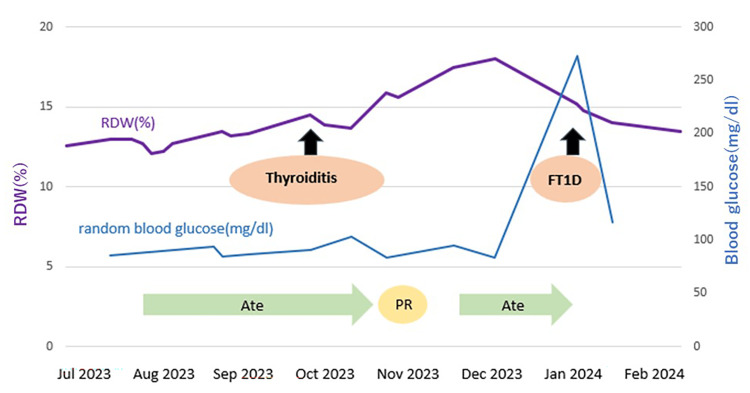
RDW and irAE onset timeline (Case 1) Two months after initiating atezolizumab (Ate), the patient developed thyroiditis, which subsequently progressed to hypothyroidism. The therapeutic response to atezolizumab was a partial response (PR). At five months, the patient was diagnosed with fulminant type 1 diabetes. The RDW began to increase two months before the onset of thyroiditis, showed a temporary decrease, and then peaked one month before the onset of fulminant type 1 diabetes. irAE: Immune-Related Adverse Events; RDW: Red Cell Distribution Width

Upon admission, intensive insulin therapy was initiated. On hospital day two, her fasting blood glucose was 339 mg/dL, and her two-hour postprandial glucose was 345 mg/dL. Fasting C-peptide immunoreactivity (CPR) was 0.39 ng/mL, two-hour postprandial CPR was 0.42 ng/mL, and 24-hour urinary CPR was 1.28 μg/day, indicating significantly impaired endogenous insulin secretion. Her insulin secretory capacity continued to decline, and by hospital day 14, serum CPR was undetectable, and urinary CPR had decreased to 0.16 μg/day, confirming substantially complete depletion of her pancreatic β-cell function. After appropriate insulin dose adjustments, she was discharged. Intriguingly, the new potential biomarker associated with aging, RDW, was transiently increased before the onset of ICI-related adverse events (Figure 2), suggesting possible involvement of aging-related biological processes in this clinical course.

Case 2

A 74-year-old Japanese man, without any history of diabetes mellitus, was diagnosed with advanced gastric cancer and esophageal cancer, and his HbA1c was found to be markedly elevated to 10.9%. During hospitalization for further evaluation, anti-GAD autoantibody was negative, and 24-hour urinary CPR was 46 μg/day, suggesting decreased insulin secretion. Based on these findings with imaging results, he was diagnosed with pancreatic diabetes secondary to pancreatic invasion by gastric cancer. Intensive insulin therapy was initiated, resulting in improvement of HbA1c to the 6% range.

Two months later, combination therapy with oxaliplatin and tegafur/gimeracil/oteracil potassium (SOX), along with nivolumab (PD-1 antibody), was initiated. The disease was subsequently assessed as stable disease (SD). Three months after initiation of nivolumab, he developed fatigue and anorexia and was subsequently diagnosed with isolated adrenocorticotropic hormone (ACTH) deficiency associated with nivolumab therapy. Hydrocortisone replacement therapy was initiated, and in part because of this adrenal insufficiency, insulin therapy was temporarily discontinued, with management switched to oral antidiabetic agents. However, his HbA1c rose to the 8% range after a while, insulin was reintroduced, and HbA1c subsequently stabilized around 7%.

**Figure 3:**
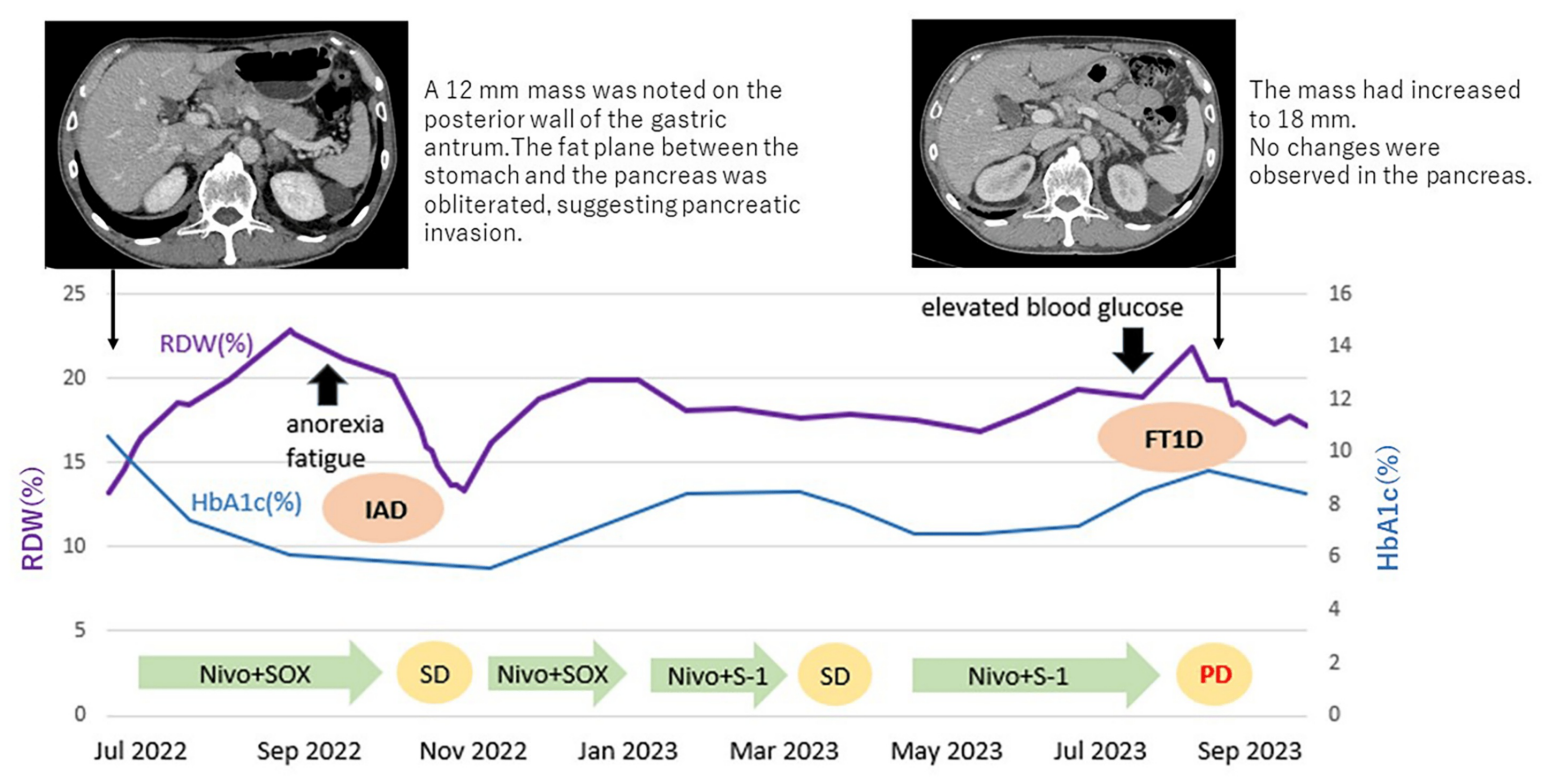
RDW and irAE onset timeline (Case 2) Three months after initiating nivolumab (Nivo), the patient was diagnosed with isolated adrenocorticotropic hormone deficiency (IAD). The therapeutic response to nivolumab was stable disease (SD). Thirteen months after treatment initiation, a sudden increase in self-monitored blood glucose (SMBG) was observed, and the patient was diagnosed with fulminant type 1 diabetes. The RDW showed an upward trend prior to the onset of ACTH deficiency, decreased temporarily, and then rose again, peaking immediately after the diagnosis of fulminant type 1 diabetes. irAE: Immune-Related Adverse Events; RDW: Red Cell Distribution Width; SOX: Oxaliplatin and Tegafur/Gimeracil/Oteracil Potassium; S-1: Tegafur/Gimeracil/Oteracil Potassium

Thirteen months after the initiation of nivolumab, he noticed a sudden and unexpected rise in blood glucose levels in his daily blood glucose monitoring. Even with increased insulin doses prescribed in the outpatient setting, glycemic control remained poor. He also experienced an unintentional weight loss of 4 kg over the course of one month and was admitted in the following month for further evaluation. Clinical data associated with the disease course are shown as follows: his outpatient clinical course (Figure 3), the self-blood glucose monitoring (SMBG) records at the time of onset (Table 2), and laboratory findings at admission (Table 3).

**Table 2:** Self-monitored blood glucose (SMBG) records before and after onset of immune checkpoint inhibitor (ICI)-induced fulminant type 1 diabetes (Case 2)

Variables	Day 3	Day 2	Day 1	Day 0	Day 1	Day 2	Day 3
Pre-breakfast blood glucose (mg/dL)	165	128	143	218	260	271	298
Pre-lunch blood glucose (mg/dL)	192	-	200	-	-	278	-
Pre-dinner blood glucose (mg/dL)	-	349	-	325	323	-	337
Post-dinner blood glucose (mg/dL)	251	230	355	-	319	-	338

**Table 3:** Laboratory data on admission (Case 2) AST: Aspartate Aminotransferase; ALT: Alanine Aminotransferase; LDH: Lactate Dehydrogenase; ALP: Alkaline Phosphatase; γGTP: Gamma-Glutamyl Transpeptidase; BUN: Blood Urea Nitrogen; FT3: Free Triiodothyronine; FT4: Free Thyroxine; TSH: Thyroid-Stimulating Hormone; GAD Antibody: Anti-glutamic Acid Decarboxylase Antibody; IA-2 Antibody: Anti-insulinoma-Associated Antigen-2 Antibody; ZnT8 Antibody: Anti-zinc Transporter 8 Antibody; HLA: Human Leukocyte Antigen

Test	Result	Reference range
White blood cells (/μL)	3800	4000-9000
Hemoglobin (g/dL)	11.3	12-16
Platelet (10^4^/μL)	11.1	15-35
AST (U/L)	33	8-38
ALT (U/L)	30	4-44
LDH (U/L)	289	124-222
ALP (U/L)	110	38-113
γGTP (U/L)	144	8-38
Amylase (U/L)	68	40-126
BUN (mg/dL)	11.4	8-20
Creatinine (mg/dL)	0.54	0.47-0.79
Sodium (mEq/L)	136	135-147
Chloride (mEq/L)	98	98-108
Potassium (mEq/L)	4.8	3.6-5.0
FT3 (pg/mL)	2.4	2.3-4.0
FT4 (ng/dL)	1.04	0.9-1.7
TSH (μIU/mL)	4.2	0.5-5.0
HbA1c (%)	9.0	4.6-6.2
Glycoalbumin (%)	39.5	11-16
Plasma glucose (mg/dL)	264	70-199
GAD antibody (U/mL)	<5.0	<5.0
IA-2 antibody (U/mL)	<0.6	<0.6
ZnT8 antibody (U/mL)	<10.0	<10.0
Urine protein	negative	
Urine glucose	4+	
Urine ketone bodies	Negative	
Urinary C-peptide (μg/day)	<0.2	
HLA	DRB1 09:01 DQB1 03:03 DPB1 05:01	

On admission, his random blood glucose was 264 mg/dL, serum CPR was 0.02 ng/mL, and urinary CPR was < 0.2 μg/day, indicating complete loss of endogenous insulin secretion. Imaging revealed progression of the primary malignancy and was assessed as progressive disease (PD); however, no new or significant changes were observed in the pancreas, indicating the first progression of impaired insulin secretion was not caused by exacerbation of the invasion in the pancreas. Based on these findings and the SMBG profile, a diagnosis of ICI-induced fulminant type 1 diabetes was made. Insulin therapy was intensified during hospitalization, and he was subsequently discharged. Similar to Case 1, we observed transient increases in RDW levels before the onset of both irAE episodes in this case, suggesting that RDW levels may be useful to predict the possible occurrence of irAEs in the near future (Figure 3).

## Discussion

We have experienced two ICI-T1D cases, which were successfully diagnosed and treated without the development of severe hyperglycemia. While typical FT1D is often found with severe hyperglycemia and ketoacidosis, our experience here, together with reported cases, highlights the differences from ICI-T1D and provides insights into the appropriate follow-up of patients undergoing ICI therapy based on these differences.

To better characterize the early clinical features of ICI-T1D with fulminant progression - particularly blood glucose levels, residual insulin secretion, and the time course of insulin depletion - we reviewed previously published cases (Table 4) [16-25]. We focused on cases diagnosed before the onset of ketosis or at a stage when insulin secretion was still preserved. Specifically, we included cases that met the following criteria: HbA1c < 8.7% at diagnosis, in addition to either absence of ketosis [5] or serum CPR ≥0.5 ng/mL [4] at onset. Cases diagnosed as acute-onset or slowly progressive type 1 diabetes were excluded, as these are generally more readily detected by routine outpatient testing. The time to insulin depletion was defined as the number of days from the first detection of hyperglycemia to the point at which insulin depletion criteria were met: urinary CPR < 10 μg/day, fasting serum CPR < 0.3 ng/mL, or random serum CPR < 0.5 ng/mL. The following analysis was conducted after excluding cases in which evaluation was difficult due to missing data or other limitations.

**Table 4:** Summary of the reported cases of immune checkpoint inhibitor-induced fulminant type 1 diabetes ICI: Immune Checkpoint Inhibitor; CPR: C-Peptide Reactivity; IRI: Immunoreactive Insulin; HLA: Human Leukocyte Antigen; irAE: Immune-Related Adverse Event; Refs: references; Pem: Pembrolizumab; Nivo: Nivolumab; Ave: Avelumab; Sin: Sintilimab; Ate: Atezolizumab; DKA: Diabetic Ketoacidosis; GAD: Anti-glutamic Acid Decarboxylase Antibody; IA-2: Anti-insulinoma-Associated Antigen-2 Antibody Sources: Refs [16-25]

No	Age/Sex	Primary Diagnosis	ICI	Duration to diabetes	HbA1c (%)	Glucose (mg/dL)	CPR (ng/mL)	IRI (μU/mL)	Ketosis	Duration to CPR depletion	Antibody	HLA	Other irAEs	Refs
1	72/M	Non-small cell lung cancer	Pem	4 months	6.4	209	2.77	-	No	18-28 days	Negative	DRB1*09:01 DQB1*03:03	No	[16]
2	67/M	Malignant melanoma	Nivo	57 days	7.1	539	2.6	8.2	Yes	-	GAD +	DRB1*08:02 DQB*03:02	Thyroiditis, IAD	[17]
3	79/M	Non-small cell lung cancer	Nivo	9 days	6.1	81	1.56	-	No	1 day	Negative	DRB1*09:01 DQB1*03:03	No	[18]
4	55/F	Malignant melanoma	Nivo	12 months	7.0	580	1.0	-	Yes	within 14 days	Negative	DRB1*04:05 DQB1*04:01	No	[19]
5	77/M	Renal cell carcinoma	Nivo	12 weeks	6.2	379	5.92	-	No	8 days	Negative	DRB1*09:01:02/12:01:01 DQB1*03:01:01/03:03:02 DPB1*05:01:01 DQA1*03:02/ 05:05	No	[20]
6	82/M	Lung squamous cell carcinoma	Pem	54 weeks	6.1	319	2.03	-	No	16 days	Negative	DRB1*12:01	No	[21]
7	76/M	Lung adenocarcinoma	Pem	1 month	6.3	616	0.81	-	-	within 14 days	GAD + IA-2 +	-	No	[22]
8	79/F	Merkel cell carcinoma	Ave	5 months	7.5	483	1.07	4.3	Yes	within 7days	Negative	DRB1*09:01:02/14:54:01 DQB1*05:02:01/03:03:02 DQA1*01:04/ 03:02	No	[23]
9	59/M	Malignant melanoma	Nivo	18 months	6.8	229	5.47	-	No	8 days	Negative	DRB1*04:06 DQB1*03:02 DRB1*04:10 DQB1*04:02	Thyroid dysfunction	[24]
10	56/M	Hepatocellular carcinoma	Sin	24 weeks	7.8	399.6	1.12	1.5	DKA	4 days	Negative	A*02:01/24:03 B*15:25/40:02 C*03:04/04:03 DRB1*12:01/ 12:02 DQB1*05:03/ 03:01 DQA1*01:04/ 06:01	No	[25]
11	75/F	Lung neuro-endocrine tumor	Ate	5 months	5.2	273	-	3.6	No	2 days	Negative	DRB1 09:01:02/13:02:01 DQB1 06:04:01/03:03:02 DPB1 04:01:01/05:01:01	Hypothyroidism	

The interval from ICI initiation to diabetes onset varied widely, ranging from nine days to 18 months, with no consistent trend observed. Hyperglycemia was first noted as random blood glucose levels ranging from 209 to 616 mg/dL. Notably, in three cases - including our case - hyperglycemia was detected at levels below the 288 mg/dL, a threshold defined in the diagnostic criteria for fulminant type 1 diabetes [7]. Serum CPR exceeded 1.0 ng/mL at the time of diagnosis in nine of 10 cases. IRI levels were above 3.0 μU/mL in three of four analyzable cases. Time to insulin depletion was within seven days in four of 10 cases - including our case - while in the remaining six cases, it was eight days or more.

These findings suggest that, even in the presence of only mild hyperglycemia and partially preserved insulin secretion, its onset should be suspected with careful attention, and in such cases, early intervention - including hospitalization for further evaluation, initiation of insulin therapy or self-monitoring of blood glucose, and close follow-up - should be strongly considered.

The onset speed of ICI-T1D has been reported to be slower than that of conventional FT1D, but more rapid than that of acute-onset type 1 diabetes [26]. Our review is consistent with this observation. Previous reports have estimated that the average time to complete insulin depletion in conventional FT1D is approximately 5.3 days [27]. In contrast, more than half of the reviewed ICI-T1D cases required eight days or longer to reach equivalent depletion, suggesting slower progression. These differences may reflect underlying pathophysiological distinctions between conventional FT1D and ICI-T1D [28]. Since most patients undergo regular blood testing during ICI treatment, careful monitoring may allow for the timely detection of ICI-T1D.

One of the unique observations in this report is the possible irAE prediction by detecting the transient increase in RDW levels. Indeed, we need a larger number of samples to achieve meaningful conclusions, and this possibility is currently under extensive investigation in our group. Importantly, most studies that analyze large numbers of samples adopt a cross-sectional design, focusing on data from a single time point. Such approaches are unable to capture transient elevations in the measured parameters. In contrast, our present study, which carefully examined the temporal course, was able to notice the potential power of the transient increase or RDW levels to predict irAEs. The finding is currently premature but warrants further investigation in the future. The transient increase in RDW levels seems to be applicable to irAEs in general, rather than specific to ICI-T1D, although the mechanisms behind this remain elusive. Similarly, the pathogeneses underlying the irAEs also remain largely unknown; however, the possible association between RDW and irAEs may offer clues to identify key mechanisms behind irAEs through investigating factors influencing erythropoiesis.

Regarding the association with HLA genotypes, previous studies have reported that HLA haplotypes commonly seen in conventional type 1 diabetes include DRB109:01-DQB103:03, DRB104:05-DQB104:01, and DRB108:02-DQB103:02, while ICI-T1D has been more frequently associated with DRB104:05-DQB104:01 [9]. In our review, among 11 cases with available HLA typing (including our Case 2), six had DRB109:01-DQB103:03, one had DRB108:02-DQB103:02, and one had DRB104:05-DQB104:01. These findings suggest that DRB109:01-DQB103:03, more commonly associated with conventional type 1 diabetes, was also frequently seen in ICI-T1D, implicating that some of the immunological backgrounds are shared by these two. This may also indicate that individuals with a genetic predisposition to autoimmune diabetes could develop ICI-T1D when exposed to immune checkpoint blockade. However, it remains unclear whether HLA type influences the clinical features of ICI-T1D.

## Conclusions

We have experienced two ICI-T1D cases successfully diagnosed and treated. Based on these cases along with the literature, we attempted to find optimal ways to follow up patients receiving ICI therapy. The course of ICI-T1D tends to be slower, and careful monitoring of glycemic metrics should be of critical importance even with mildly elevated blood glucose levels and partially preserved insulin secretion. Moreover, RDW levels may be worth consideration as a predictive marker of irAEs.
